# VLMs struggle to extract definitions of structural segments from MSA images: toward the design of a human-in-the-loop annotation pipeline for the PDB-Descriptome project

**DOI:** 10.1186/s44342-025-00061-4

**Published:** 2026-03-26

**Authors:** Koya Sakuma

**Affiliations:** 1 Cellular and Structural Physiology Institute, Nagoya University, Furo-cho, Chikusa-ku, Nagoya, 464-8601 Japan; 2 Graduate School of Pharmaceutical Sciences, Nagoya University, Nagoya, Japan

**Keywords:** Structural Biology, Vision-language models, Multiple sequence alignment, Referring expression comprehension, Molecular structure annotation, PDB-Descriptome

## Abstract

We investigate the ability of vision–language models (VLMs) to extract structural region definitions from rasterized multiple sequence alignment (MSA) images, as part of the PDB-Descriptome project. Using synthetic MSAs with annotated structural biological entities (SBEs) and structural biological Referring Expressions (SBREs), we evaluate two VLMs, gemini-2.5-flash and gemini-2.5-pro, under naïve and strict prompts. While VLMs perform well in SBRE extraction, they show poor accuracy in defining SBE boundaries. In contrast, a human annotator achieves high boundary precision with slightly lower textual accuracy. These results support a human-in-the-loop pipeline for reliable structure–text annotation.

**Supplementary Information:**

The online version contains supplementary material available at 10.1186/s44342-025-00061-4.

## Background

### Structural biology and biomolecular structure data

Structural biology addresses biological questions from a microscopic perspective by determining the three-dimensional structures of the molecules involved [1]. Virtually all publications on experimental structure determination in this field are linked to at least one biomolecular structure deposited in the Protein Data Bank (PDB) [2–4]. Typically, biomolecular structure data comprise a set of three-dimensional coordinates of atoms, atom names, residue names, and their indices; it is formatted in a machine-readable format such as mmCIF [5]. Based on the structures, structural biologists often describe not only the overall shape of a molecule but also specific segments that are functionally relevant. The descriptions range from the entire architecture of biomolecular complexes to atomistic details such as hydrogen bonds and electronic states. Consequently, structural biology literature is inherently multimodal, combining the conventional format of scientific papers with three-dimensional molecular structure data.

Despite the long history of structural biology and the standardization of data for biomolecular structures, the linkage between textual descriptions in the literature and molecular structures described there has never been systematically standardized. The PDB-Descriptome, introduced in BLAH9, is an ongoing project to address this issue by establishing a framework to systematically link 3D biomolecular structures with detailed descriptions [6, 7]. The project aims to create a novel class of multimodal data systems through the development of consistent annotation rules, data formats, and supporting tools to accelerate annotation. The resultant paired dataset of structures and descriptions is expected to enable linking structures and their meanings, leading to advanced data science efforts to model the act of structural biology itself.

### Structural biological entities and structural biological Referring Expressions

The absence of explicit mappings between referring expressions in the text domain and their definitions in the structure domain is the primary barrier to integrating literature with molecular structure data. For example, a paper might describe “the catalytic domain” or “the active site” without providing a precise mapping such as “the catalytic domain denotes residues 41–125 and 199–230” or “residues 50, 54, and 201 constitute the active site.” The PDB does not have a system to compensate for this lack. In this project, we introduce two dedicated terms to articulate the specific tasks to be solved: *structural biological entities* (*SBEs*) in the structure domain and *structural biological referring expressions* (*SBREs*) in the text domain. The former denotes what is referred to, and the latter denotes what performs the referring.

In the structural data domain, we term the substructures in the original whole structure SBEs. In typical atomic coordinate-based data, SBEs can be defined as sets of atom indices. SBEs are arbitrary substructure definitions and can be considered analogues to segment information used to extract specific regions in an image. Relevant SBEs can be the subjects of the corresponding structural biology papers to describe the features of the molecule in natural language and figures. Although SBEs might extend to or span between multiple structures (e.g., discussions involving structure comparisons), we focus on cases involving a single PDB entry in this paper.

In the corresponding publications, SBEs are referred to in various ways. Typically, if the description focuses on specific residues, SBEs are referred to by combinations of residue names and residue indices, such as “Glutamic acid residue at the 81st site,” “Glu81,” “E81,” or even just “residue 81.” This can be extended to larger substructures like “the fifth alpha-helix” or “H5.” Similarly, when the description is more specific to functional group- or atom-level features, they are referred to as such for example, “the side chain carbonyl group of Glu81” or even just “the carbonyl group,” depending on the context. In addition, authors sometimes give “nicknames” to SBEs to make them more human-friendly if the SBE is a more composite substructure, such as domains, specific groups of secondary structures, or groups of residues like active sites. Such nicknames tend to be defined locally in the specific literature but may eventually be adopted across the field.

Given the domain-specific nature of these referring expressions, we term all the words, sets of words, or phrases that refer to SBEs in the text domain SBREs. These are typically represented by spans in the text domain. Importantly, general SBREs such as “Glu81,” “H5,” and “the carbonyl group” can frequently appear in other literature for unrelated structures and cannot be assumed to refer specifically to the structure of interest. Nickname-like ones are neither general nor exclusive among publications. Therefore, in either case, it is nearly meaningless to treat the referring expressions as isolated textual data, and thus it is essential to link SBREs in the text domain to their corresponding SBEs in the structural domain.

### Scope of SBEs and SBREs extraction from literature

Currently, the project targets PDB entries and the structural biology literature that describes them as primary data sources. Structural biology papers often define pairs of SBEs and SBREs in context, and typical approaches to define SBE–SBRE pairs can be classified into three types: (1) immediate introduction of SBREs in the text; (2) defining in sequence-related figures and their legends; (3) defining in structure-related figures and their legends. Other common cases may borrow them from previous papers, which can be solved by combining with analysis of referenced literature if we have scalable solutions to resolve self-contained cases.

In this work, we focus on a simplified case of (2) and investigate the ability of vision–language models (VLMs) to extract SBEs and SBREs from raster images presenting MSAs. In the situation of (2), SBEs and SBREs are defined in figures to describe sequence features of the molecule, such as raw amino acid sequences and multiple sequence alignments (MSAs). This might directly provide information to identify which SBEs, in terms of residue indices, can be mapped to specific SBREs. While MSAs are originally machine-readable, they are often visualized as raster images in biological papers, which makes data extraction more difficult. Efforts to solve the cases of (1) and (3) will be reported elsewhere.

## Methods

### Synthetic data generation

**Fig. 1:**
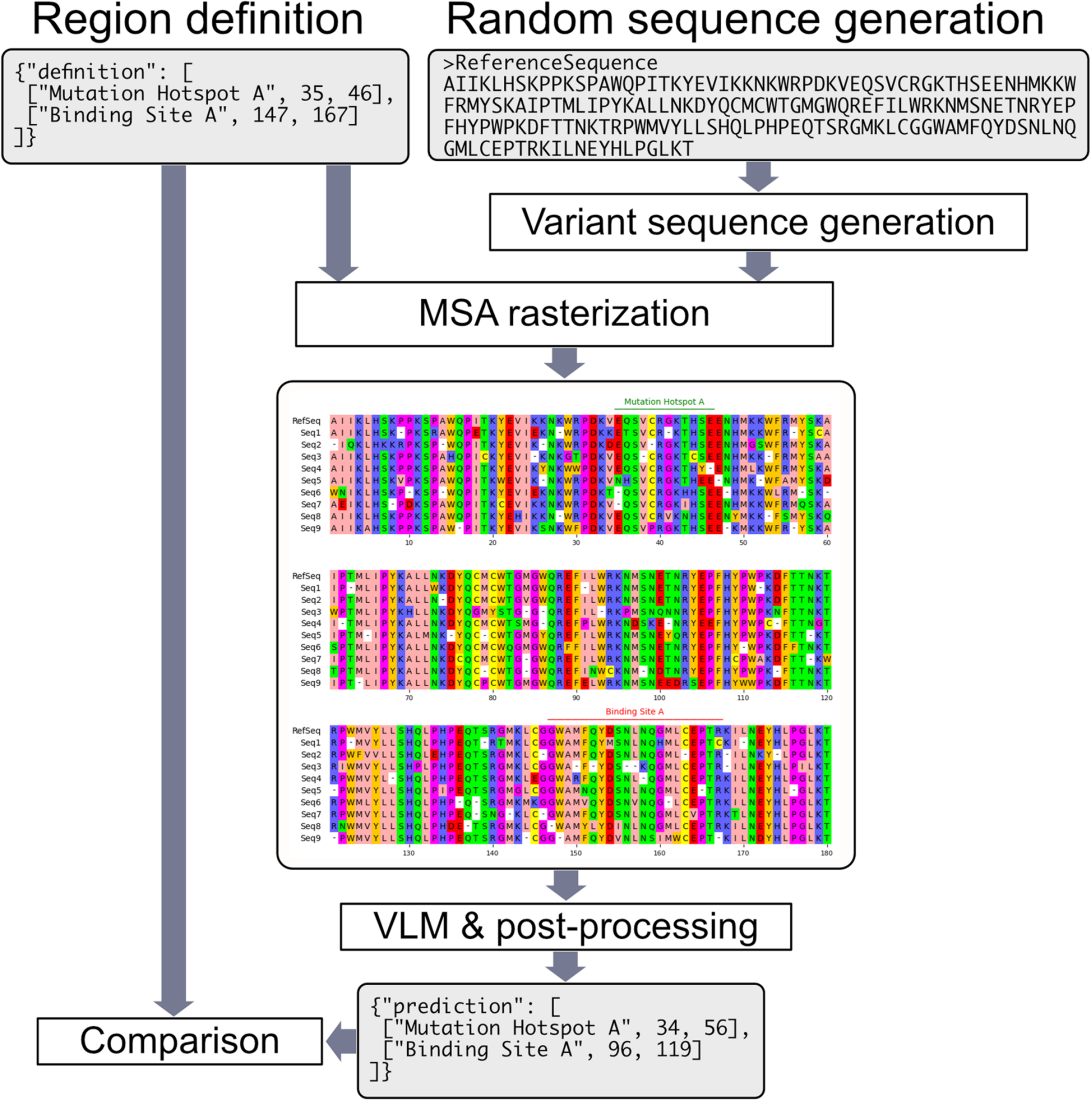
Schematic diagram of the evaluation pipeline based on synthetic MSA images Synthetic MSA images were generated based on random sequences and region definitions. The MSA images were analyzed by VLMs and their responses were compared with the ground truth region definitions

To evaluate the mask and region name extraction capabilities of multimodal large language models (LLMs), particularly vision–language models (VLMs), synthetic MSA images generated from artificial amino acid sequences and mask data were used instead of real images from published papers (Fig. 1). This approach eliminated the need to manually create ground-truth data and enabled simplified testing.

A synthetic amino acid sequence of 180 residues was first generated by randomly selecting residues with replacement from the 20 standard amino acids (ARNDCQEGHILKMFPSTWYV). Nine additional child sequences were then created by introducing mutations and gaps with probabilities of 0.1 and 0.05, respectively, resulting in a total of ten sequences including the parent. A synthetic MSA was constructed from these sequences.

For each MSA, between two and five regions were randomly assigned. Each region was given a name such as “Mutation Hotspot,” “Low Conservation,” “Binding Site,” or “Structural Motif,” with unique identifiers appended (A, B, C, …) to ensure distinct naming. The length of each region was randomly chosen between 10 and 30 residues, and placement was randomized with a requirement for at least four-residue gaps to prevent overlap. Across the 100 datasets, this procedure produced a total of 377 regions.

**Fig. 2:**
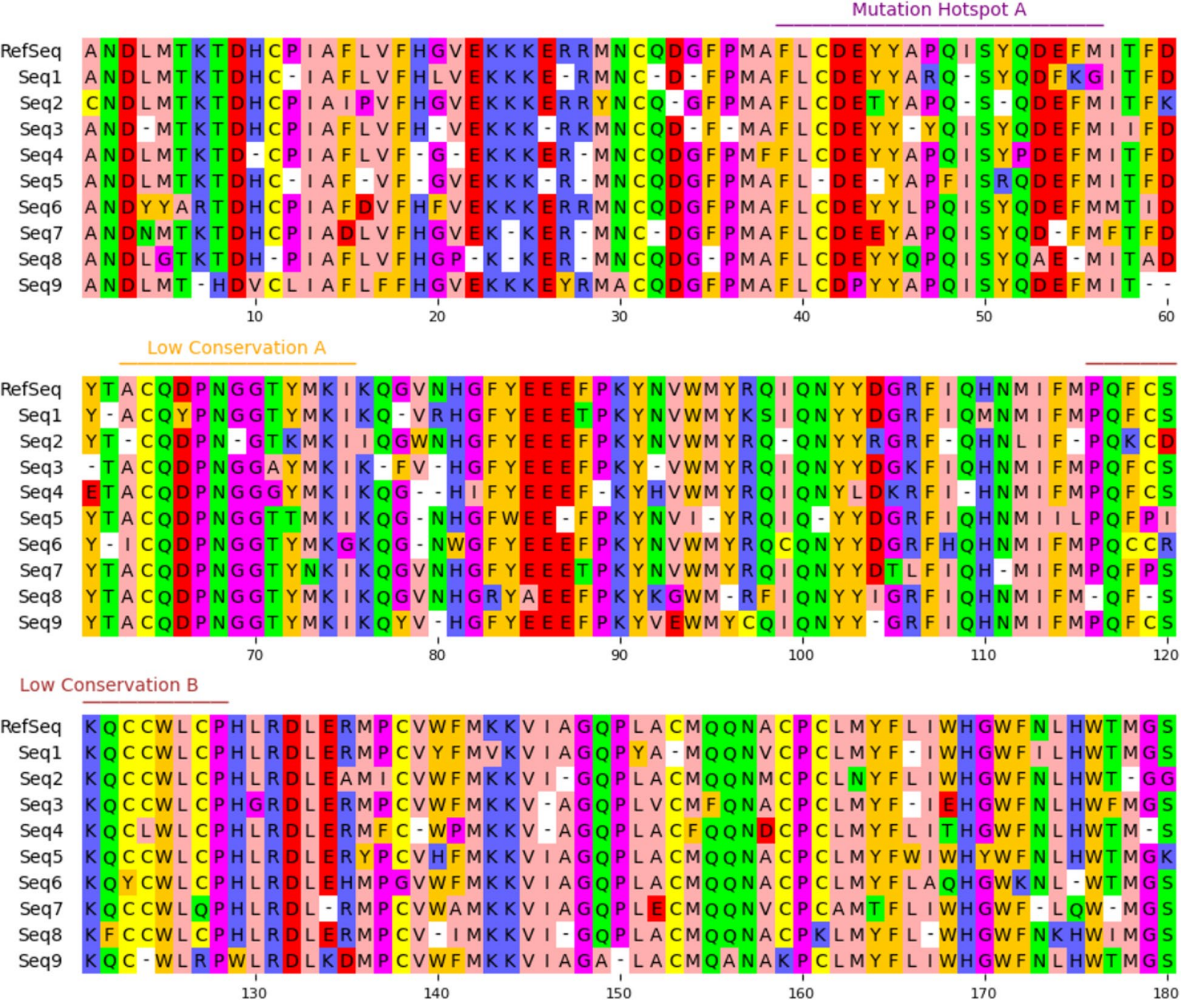
Example synthetic MSA image The image shows a multiple sequence alignment (MSA) of 10 sequences with annotated regions labeled as SBREs (e.g., “Mutation Hotspot A,” “Low Conservation A,” and “Low Conservation B”). Colored bars and text above the alignment indicate the names and positions of the annotated structural regions. Readers need to extract SBREs and estimate residue indices to define SBEs. The source annotation data is identical to that in Table 1

The pyMSAviz library [8] was used for visualization of the MSAs with the region annotations. Residue numbers were displayed at intervals of 10, and sequences were broken into lines every 60 residues. Regions were indicated by bars colored randomly in red, green, blue, orange, or purple, with the region names placed above in the same color as the bars. Each dataset, consisting of the synthetic MSA with its region annotations, was exported as a raster image in PNG format with a resolution of 1006 × 867 pixels. In total, 100 such images were generated. An example MSA image and its ground truth annotations are shown in Fig. 2 and Table 1.

**Table 1:** Ground truth annotations for the synthetic MSA image

SBRE	SBE definitions	
	**Start**	**End**
Mutation Hotspot A	39	56
Low Conservation A	63	75
Low Conservation B	116	128

The source annotation data is identical to that in Fig. 2

Although the use of ten fixed-length sequences is not realistic compared with real-world scenarios where MSAs vary across papers, this setup was considered sufficient to estimate an approximate upper bound on capabilities, since it is unlikely that performance would improve with more diverse data.

### VLM selection and prompting

For the extraction task, gemini-2.5-flash and gemini-2.5-pro were used as the subject VLMs. Two types of prompts, naïve and strict, were prepared (shown below) and submitted to gemini-2.5-flash together with the synthetic MSA images. For gemini-2.5-pro, only the strict prompt was used.

#### Naïve prompt

This is a figure from a biological paper. Identify any structural region defined here. Report the python list of lists as parsed_result = [[region_name, region_init, region_end],...]

#### Strict prompt

This is a figure from a biological paper. Identify any structural region defined here. You must extract the residue indexes as precisely as possible from the figure, ideally at one-residue resolution. Residue indexes are shown at sparse, roughly regular intervals for clarity, so you'll need to estimate the actual start and end positions of structural regions by interpolating between the visible labels. Report the python list of lists as parsed_result = [[region_name, region_init, region_end],...]

### VLM output handling

The raw response was stripped of formatting characters, and the portion following the predefined output key (i.e., “parsed_result = ”) was extracted. If parsing into a Python list succeeded, the result was immediately returned. If parsing failed due to missing keys or syntax errors, the model was queried again, up to three times. If all three attempts failed, a blank result was returned.

### Human annotation

To establish a reference for comparison with the VLM-based extraction, human annotation was conducted on the same set of synthetic MSA images, which were displayed on a 13-inch laptop screen. A trained structural biologist (single annotator) was asked to identify all structural regions in each figure and to record both the region names and their corresponding residue ranges. No time limit was imposed on the annotation task. For text input, Microsoft Excel was used, and the annotator was allowed to use text auto-completion to replicate the same or similar SBRE input from preceding MSAs. The annotator was also permitted to use a ruler without tick marks to assist with boundary estimation.

### Evaluation

Evaluation of the outputs from the VLMs and the human annotator was carried out at two levels. For region name extraction (i.e., SBRE extraction), the lists of extracted strings were converted into sets to remove duplication for each individual MSA image, and each string was counted as correct only when it exactly matched the ground truth. Precision, recall, and F1-score were then calculated over all MSA images. Precision was defined as the number of correct extractions divided by the total number of extractions, and recall was defined as the number of correct extractions divided by the total number of ground-truth extractions.

For region boundary extraction (i.e., numerical definitions of SBEs), the differences between the extracted residue numbers and the ground-truth values were calculated. In cases where the region names did not match correctly or were duplicated, the corresponding numerical data were excluded from the evaluation.

## Results

### General observations

**Fig. 3:**
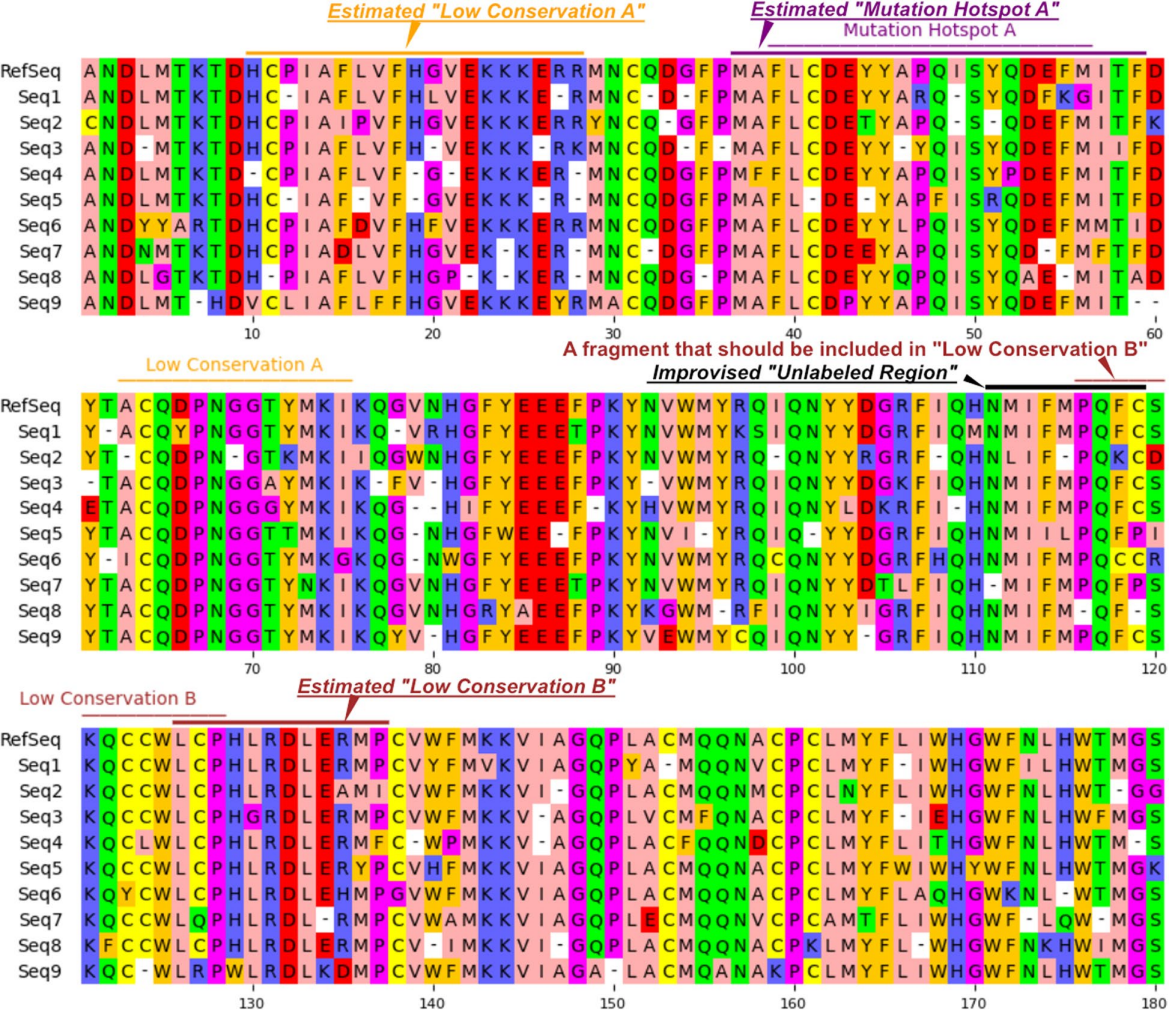
Comparison between VLM-based annotations and the original MSA image The source MSA image is the same as in Fig. 2. Regions of SBEs estimated by a VLM (gemini-2.5-pro) are indicated by bars with SBREs in italics, colored similarly to the ground truths. Residues 111–119, which should have been included in “Low Conservation B,” were misrecognized as an independent region and given the improvised name “Unlabeled Region.” The source annotation data is identical to that in Table 2

The pipeline with gemini-2.5-flash successfully processed all the MSA images within three retries, while gemini-2.5-pro failed to produce a parsable response in 6 cases out of 100. Table 2 and Fig. 3 show example VLM-based (gemini-2.5-pro) annotations from a synthetic MSA image. This example was selected since it includes a near-correct extraction, a row-swapping mistake, and improvised labels.

**Table 2:** Example extraction from gemini-2.5-pro with an improvised region name

SBRE	SBE definitions	
	Start	End
Mutation hotspot A	37	59
Low conservation A	10	28
Low conservation B	126	137
Unlabeled region	111	119

The source annotation data is identical to that in Fig. 3

Interestingly, although the prompt explicitly specified the output format, unparsable responses from gemini-2.5-pro sometimes included Python code seemingly designed to solve the extraction task or to return the list content using a print function (Supplementary text S1 and Table S1), which the downstream parser was not designed to handle. The 6 failed cases appear to have resulted from three unparsable outputs in a row.

The human annotator reported several observations: only the first approximately 20 MSAs were processed using the ruler, with the remainder inspected visually; the color coding of the synthetic MSAs was somewhat distracting, with bars and characters in certain colors being especially difficult to distinguish due to low contrast against the white background; some loss of focus occurred during the final approximately 20 MSAs; and typographic errors could have been more frequent if autocompletion had not been allowed.

### Annotation time

For the full set of 100 synthetic MSA images, gemini-2.5-flash required approximately 20 min, while gemini-2.5-pro required 2 h and 30 min. Since five-second pauses were added between each MSA image to avoid exceeding the Gemini API quota, and three-second pauses were added before retries, this execution time could in principle be shorter. The human annotator required about 2 h and 10 min under the no-time-limit condition.

### Extraction accuracy

All outputs from VLM annotation with naïve prompting, VLM annotation with strict prompting, and human annotation, respectively, were evaluated in the same manner. Precision, recall, and F1-score were calculated for extracted SBREs, and mean absolute errors (MAEs) from the ground-truth values were calculated for the numerical definitions of SBE boundaries. Specifically, separate MAEs were computed independently for the start and end residue indices of each region. Results are summarized in Table 3.

**Table 3:** Precision, recall, F1-score, and mean absolute errors (MAE) for SBE and SBRE extraction by VLMs and a human annotator

	Gemini 2.5 Flash (naïve)	Gemini 2.5 Flash (strict)	Gemini 2.5 Pro (strict)	Human annotator
Precision	0.997	1.000	0.994	0.987
Recall	1.000	1.000	0.944	0.984
F1-score	0.999	1.000	0.969	0.985
MAE (start)	13.642	12.627	8.921	0.113
MAE (end)	13.795	12.603	9.576	0.278

While we observed that gemini-2.5-flash sometimes extracted the same SBRE multiple times, both precision and recall remained high. The precision and recall of gemini-2.5-pro were lower than those of gemini-2.5-flash because of the unintended code-generation responses described in Section 3.1 and the improvised segment names assigned to fragmented regions spanning multiple rows (e.g., “Unlabeled Region” and “Unknown”) (Fig. 3).

**Fig. 4:**
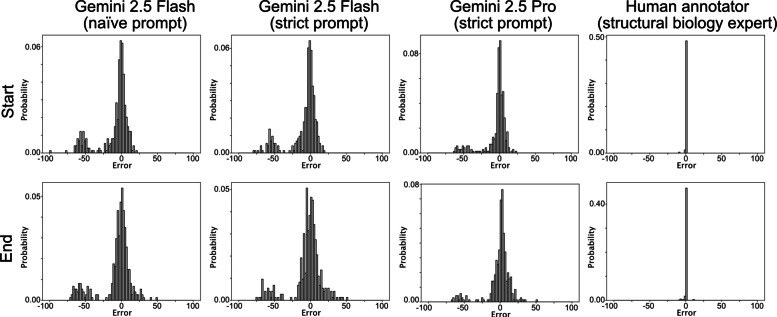
Distribution of errors in estimated boundary residue indices The vertical axis corresponds to the errors between estimated values from MSA images and the ground truths. Error distributions for start residues are shown in the upper panels and for end residues in the bottom panels

The MAEs of VLM-extracted boundaries were large, effectively spanning regions corresponding to an entire row of the MSA. The strict prompt slightly reduced these errors for gemini-2.5-flash, and gemini-2.5-pro showed better accuracy in SBRE extraction. Compared with the VLMs, the human annotator showed slightly lower precision and recall, which can be attributed to typographic errors. The MAEs of human-extracted boundaries were strikingly smaller than those of the VLMs. The histograms of raw error values show that the human annotator was clearly superior to the VLMs (Fig. 4). While VLM-based extractions were broadly distributed around zero, additional peaks around − 60 were observed in the VLM outputs. These additional peaks can be attributed to misassignment of the region-indicating bars to the wrong rows, especially to one row above the ground truth.

## Discussion

The VLMs demonstrated reasonable performance overall, but clear differences emerged in the orientations of VLM and human abilities for SBE and SBRE extraction. In particular, extracting the numerical definitions of SBEs posed a substantial challenge for the VLMs. In contrast, human annotation achieved nearly two orders of magnitude greater accuracy in boundary extraction, suggesting that a human-in-the-loop strategy would be a practical solution for accelerating the construction of high-quality structure–text paired datasets. In this light, it will be important to further evaluate the abilities of non-expert human annotators, including the inter-annotator consistency of annotations, and to consider methods for training them. While our current analysis focused on the execution time, the cost of compensation for professional human annotators should be evaluated against the cost required to realize the automation.

Regarding the unparsable responses from gemini-2.5-pro containing Python code, improved prompt design and more robust parsers could help achieve higher reliability. For example, negating instructions such as “Do not generate code.” might improve the response, but it is difficult to anticipate and prevent all possible irregular outputs. Therefore, another possible approach would be to incorporate a secondary instance of a VLM or LLM to validate whether the response conforms to the requirements and to reformat it if necessary before it is passed to the parser. In addition, the “intentions” of VLMs, such as those found in the unparsable responses from gemini-2.5-pro, suggest how the VLM interprets MSA images and may provide useful insights for refining prompts and designing supplementary pre-processing protocols for input images (e.g., adding grids and interpolated numbers to images). Better prompts could possibly improve the accuracy, as indicated by the comparison between naïve and strict prompts; however, we expect the observed struggles of VLMs on the present task should be attributed to intrinsic limitations, such as the model architecture, training objectives, or the fundamental challenge of mapping visual features to precise numerical coordinates in rasterized images.

It should be noted that informal tests using ChatGPT 5.1 (Instant and Thinking) and Claude Sonnet 4.5 through their chat-based GUIs on a handful of actual MSA images extracted from structural biology papers showed similar tendencies to what systematic experiments for Gemini clarified; they performed reasonably well on SBRE extraction but struggled with accurate boundary detection (data not shown). In fact, this observation motivated us to design systematic evaluation using synthetic data. Interestingly, ChatGPT 5.1 Thinking demonstrated a promising direction; it tried the iteration of cropping images, refinement of cropped regions, reasoning based on the x–y coordinates for cropping, and mapping the x–y coordinates to residue numbers using custom Python codes. However, actual improvement of the accuracy was limited, and the execution required more than 25 minutes for a single image. Given these preliminary results, a comprehensive comparison across multiple VLM families using our controlled experimental framework would be valuable future work to determine whether the observed boundary extraction limitations are specific to Gemini or represent a broader challenge across current VLMs.

To pursue further automation, two avenues may be considered: developing dedicated extraction tools or fine-tuning existing VLMs with synthetic MSA training data. For example, boundary extraction could be facilitated by augmenting synthetic MSAs with gridlines or explicit *x*–*y* coordinate labels, which could be interpreted more robustly by the model or by auxiliary parsers. A key challenge, however, will be to account for the visual and representational heterogeneity present in published MSAs, such as differences in column width, tick frequency, and indexing conventions. Given this heterogeneity, the current results for synthetic MSA images likely represent the upper limit of accuracy; we expect worse and more inconsistent results for realistically heterogeneous MSA representation styles.

Although this study focused on parsing MSA images, the extraction or inference of SBEs and SBREs from structural figures themselves remains an even greater challenge. Such figures are highly complex and stylistically variable, making them far more difficult to process than MSAs. Synthetic data approaches may still provide a path forward, but the task will be inherently more demanding. In addition, while SBEs and SBREs are typically defined in text, sequence-related images, and structure-related images, other sources of information can also be identified. It is therefore important to consider both the ideal and the sufficient levels of coverage of SBE–SBRE pairs to be extracted from structural biology publications. To balance scalability and reliability of annotation, it is essential to establish consistent rules for what should be annotated in the PDB-Descriptome project.

## Conclusions

We evaluated VLM- and human-based extraction of SBEs and SBREs from synthetic MSA images as a minimal case study in automated analysis of structural biology papers, aimed at scaling up the PDB-Descriptome project. The results showed that the VLMs performed well in SBRE extraction but poorly in SBE boundary definition, whereas the human annotator showed the opposite profile. These findings support a practical human-in-the-loop pipeline, in which SBRE extraction is delegated to VLMs and SBE extraction is performed by humans. Future advances in VLMs may reduce the need for human intervention, but for now hybrid approaches are the most reliable.

## Supplementary Information

Supplementary Material 1: Supplementary text S1. An example response including Python code from gemini-2.5-pro. Table S1. Comparison of execution output from the code in Supplementary text S1.

## Data Availability

Synthetic MSA data and Python scripts used to replicate this study can be provided upon request to the author.

## Abbreviations

PDB: Protein Data Bank
SBE: Structural Biological Entity
SBRE: Structural Biological Referring Expression
MSA: Multiple Sequence Alignment
LLM: Large Language Model
VLM: Vision–Language Model
MAE: Mean Absolute Error
