# Supplementary material for: VLMs struggle to extract definitions of structural segments from MSA images: toward the design of a human-in-the-loop annotation pipeline for the PDB-Descriptome project
Source: Genomics Inform. 2026 Mar 26;24:6. doi: 10.1186/s44342-025-00061-4 (PMC13091262; doi:10.1186/s44342-025-00061-4)
Supplement: Supplementary file 1 — Supplementary Material 1: Supplementary text S1. An example response including Python code from gemini-2.5-pro. Table S1. Comparison of execution output from the code in Supplementary text S1. [file 44342_2025_61_MOESM1_ESM.docx]

**Supplementary Materials for “VLMs Struggle to Extract Definitions of Structural Segments from MSA Images: Toward the Design of a Human-in-the-Loop Annotation Pipeline for the PDB-Descriptome Project” by Koya Sakuma**

**Supplementary Text S1: An example response including Python code from gemini-2.5-pro.**

```python

import re

def parse_msa_regions(image_path):

"""

This function is a placeholder for a real image analysis pipeline.

For this task, the analysis is done manually by inspecting the image.

The identified regions and their coordinates are hardcoded below.

"""

# Manually identified regions from the image.

# The process involves:

# 1. Identifying the labels for each structural region (e.g., "Structural Motif B").

# 2. Locating the start and end of the horizontal bars that define these regions.

# 3. Using the residue index numbers at the bottom of the alignment to determine the exact start and end residue positions.

# 4. Interpolating between the provided index labels to find the precise one-residue index for the start and end of each region.

# Detailed breakdown:

# - The residue numbering starts at 1.

# - Labels are provided every 10 residues (10, 20, 30, ...).

# - By counting residues from the nearest label, we can determine the exact start and end positions.

# Structural Motif B:

# - Starts at the very first residue, which is index 1.

# - Ends exactly on the column labeled '30'.

# - Range: 1-30.

# Structural Motif A:

# - Starts immediately after residue 30, so at index 31.

# - Ends exactly on the column labeled '60'.

# - Range: 31-60.

# Binding Site A:

# - Starts immediately after residue 60, so at index 61.

# - Ends exactly on the column labeled '120'.

# - Range: 61-120.

# Binding Site B:

# - Starts immediately after residue 120, so at index 121.

# - Ends exactly on the column labeled '140'.

# - Range: 121-140.

# Structural Motif C:

# - Starts immediately after residue 140, so at index 141.

# - Ends exactly on the column labeled '180', which is the last residue shown.

# - Range: 141-180.

parsed_result = [

['Structural Motif B', 1, 30],

['Structural Motif A', 31, 60],

['Binding Site A', 61, 120],

['Binding Site B', 121, 140],

['Structural Motif C', 141, 180]

]

return parsed_result

# This is a dummy call to the function as the image path is not used

# The result is based on the manual analysis described above.

parsed_result = parse_msa_regions('dummy_path.png')

# Print the final result

print(parsed_result)

```

**Table S1: Comparison of execution output from the code in Supplementary Text S1**This shows modest alignment with the ground truth boundaries, which could be considered neither better nor worse than the parsable response.

|  | **SBE definitions** | | | |
| --- | --- | --- | --- | --- |
|  | **Ground truth** | | **Code ouput** | |
| **SBRE** | **Start** | **End** | **Start** | **End** |
| Structural Motif A | 41 | 65 | 31 | 60 |
| Structural Motif B | 3 | 30 | 1 | 30 |
| Binding Site A | 95 | 111 | 61 | 120 |
| Structural Motif C | 145 | 166 | 141 | 180 |
| Binding Site B | 118 | 133 | 121 | 140 |
